# Radiomics Feature Stability in True and Virtual Non-Contrast Reconstructions from Cardiac Photon-Counting Detector CT Datasets

**DOI:** 10.3390/diagnostics14222483

**Published:** 2024-11-07

**Authors:** Luca Canalini, Elif G. Becker, Franka Risch, Stefanie Bette, Simon Hellbrueck, Judith Becker, Katharina Rippel, Christian Scheurig-Muenkler, Thomas Kroencke, Josua A. Decker

**Affiliations:** 1 Department of Diagnostic and Interventional Radiology, University Hospital Augsburg, Stenglinstr. 2, 86156 Augsburg, Germany; 2 Centre for Advanced Analytics and Predictive Sciences (CAAPS), University of Augsburg, Universitätsstr. 2, 86159 Augsburg, Germany

**Keywords:** radiomics, photon-counting detector CT, virtual non-contrast

## Abstract

**Objectives**: Virtual non-contrast (VNC) series reconstructed from contrast-enhanced cardiac scans acquired with photon counting detector CT (PCD-CT) systems have the potential to replace true non-contrast (TNC) series. However, a quantitative comparison of the image characteristics of TNC and VNC data is necessary to determine to what extent they are interchangeable. This work quantitatively evaluates the image similarity between VNC and TNC reconstructions by measuring the stability of multi-class radiomics features extracted in intra-patient TNC and VNC reconstructions. **Methods**: TNC and VNC series of 84 patients were retrospectively collected. For each patient, the myocardium and epicardial adipose tissue (EAT) were semi-automatically segmented in both VNC and TNC reconstructions, and 105 radiomics features were extracted in each mask. Intra-feature correlation scores were computed using the intraclass correlation coefficient (ICC). Stable features were defined with an ICC higher than 0.75. **Results**: In the myocardium, 41 stable features were identified, and the three with the highest ICC were glrlm_GrayLevelVariance with ICC3 of 0.98 [0.97, 0.99], ngtdm_Strength with ICC3 of 0.97 [0.95, 0.98], firstorder_Variance with ICC3 of 0.96 [0.94, 0.98]. For the epicardial fat, 40 stable features were found, and the three highest ranked are firstorder_Median with ICC3 of 0.96 [0.93, 0.97], firstorder_RootMeanSquared with ICC3 of 0.95 [0.92, 0.97], firstorder_Mean with ICC3 of 0.95 [0.92, 0.97]. A total of 24 features (22.8%; 24/105) showed stability in both anatomical structures. **Conclusions**: The significant differences in the correlation of radiomics features in VNC and TNC volumes of the myocardium and epicardial fat suggested that the two reconstructions may differ more than initially assumed. This indicates that they may not be interchangeable, and such differences could have clinical implications. Therefore, care should be given when selecting VNC as a substitute for TNC in radiomics research to ensure accurate and reliable analysis. Moreover, the observed variations may impact clinical workflows, where precise tissue characterization is critical for diagnosis and treatment planning.

## 1. Introduction

Radiomics is a field of medical imaging that extracts features from images via data-characterization algorithms [1,2]. They can be used on their own or combined with other data—such as patient demographics, histology, genetics, or protein profiles—to enhance clinical decision-making and problem-solving [3]. Pyradiomics is an open-source package providing a standardized way of computing such features in different medical imaging acquisitions [4], such as computed tomography (CT) cardiac datasets [2,5,6]. CT texture analysis allows objective assessment of lesion and organ heterogeneity beyond what is possible with subjective visual interpretation and may reflect information about the tissue microenvironment [7]. Recently, recommendations have been provided to improve the quality and reliability of radiomics research [8,9].

The analysis of the myocardium and epicardial adipose tissue (EAT) in cardiac CT imaging is often performed in both non-and contrast-enhanced scans [10,11]. Photon counting detector CT (PCD-CT) systems intrinsically enable the reconstruction of virtual non-contrast data from contrast-enhanced cardiac spectral acquisitions. VNC reconstructions obtained from spectral PCD-CT data have the potential to replace native unenhanced scans in clinical routine [12]. This helps reduce the number of acquisitions and the harmful ionizing dose in comparison to energy-integrating CT systems [13]. However, the image features of true and virtual non-contrast acquisitions differ due to higher signal and contrast-to-noise ratio in virtual monoenergetic data [14]. This is especially critical for image-based computer-aided solutions [15], which rely on the extraction of features from CT non- and contrast-enhanced acquisitions [16,17]. Until now, there remains a knowledge gap in understanding the quantitative differences between TNC and VNC reconstructions and their impact on radiomics feature stability.

In this study, we, therefore, aim to quantitatively assess the interchangeability of the TNC and VNC series of EAT and the myocardium by correlating radiomics features in all series and volumes.

## 2. Materials and Methods

### 2.1. Study Population

The institutional review board (LMU Munich, project number 22-0456), with a waiver for written informed consent, approved the protocol for this retrospective single-center study. Consecutive patients with a clinically indicated ECG-gated CT scan of the heart on a PCD-CT (NAEOTOM Alpha, Siemens Healthineers, Forchheim, Germany) between January 2022 and December 2022 were included. Inclusion criteria were (1) age > 18 years, (2) pre-contrast TNC series for calcium scoring and contrast-enhanced coronary CT angiography CTA (CCTA) series, and (3) availability of raw CT data for image reconstructions.

### 2.2. Image Acquisition (Image Protocol)

All patients received a pre-contrast scan for calcium scoring followed by a CCTA at both 120 kV and a collimation of 144 × 0.4 mm. The reference tube current time product was adjusted by setting the image quality level to 19 for TNC and 60 for CTA. For the CTA, a triphasic contrast injection protocol following a test bolus was used. In the first phase, 60 mL of nonionic iodinated contrast material (Iopromide 300 mgI/mL, Ultravist, Bayer, Whippany, NJ, USA) was injected, followed by a 50% diluted mixture of 30 mL contrast material and 30 mL normal saline solution and a saline chaser (60 mL). A flow of 6 mL/s was used in all three phases. If there was no clinical contraindication, 0.4 mg of nitroglycerin was administered sublingually 5 min prior to the scan, and 5 mg of metoprolol was administered intravenously in patients with a heart rate of more than 70 bpm [12].

### 2.3. Image Reconstruction

All reconstructions were performed on a dedicated research workstation (ReconCT, Version 15.0.58331.0, Siemens Healthineers) [12]. For all patients, a TNC series based on the pre-contrast raw data and a VNC series based on the CTA were reconstructed, all at a virtual monochromatic level of 70 keV. For all reconstructions, a quantitative kernel Qr36 with a quantum iterative reconstruction calcium-preserving (VNCPC) algorithm with strength level 3 and a slice thickness/increment of 3.0/1.5 mm was used. Emrich et al. recently provided a detailed description of the VNC algorithm [18].

### 2.4. Radiomics Features Extraction

The proposed study adheres to the CheckList for EvaluAtion of Radiomics research (CLEAR) [8].

The proposed method comprises two steps: (i) Definition of volumes of interest in which Pyradiomics features are extracted, (ii) and computation of intraclass correlation values for each feature in intrapatient acquisitions.

The first step of our study consisted of segmenting myocardium and epicardial fat in TNC and VNC reconstructions of each patient. To segment the myocardium, the TotalSegmentator was utilized in Python [19]. This method is based on the nn-Unet models [20], which were pre-trained on full-body contrast and non-contrast CT acquisitions to segment multiple organs and anatomical structures (104 in total). To segment the epicardial fat, the heart mask was first obtained on a dedicated workstation (Syngo.via, version VB70A_CUT; Siemens Healthineers, using the CT Cardiac Risk Assessment application). Then, to segment the epicardial fat within a heart volume, the lower and upper thresholds were respectively set to −190 HU and −30 HU. The authors in [12] demonstrated how these threshold values on VNC series derived from PCD-CCTA datasets are accurate in segmenting epicardial adipose tissue with only minimal differences from the TNC series. A visual inspection of the generated masks was conducted by a board-certified radiologist with 5 years of experience in cardiac imaging (J.A.D.) to ensure the good quality of the segmentation. Radiomics features were extracted in the volumes of interest (VOI) using the Pyradiomics software (version 3.0.1, https://pyradiomics.readthedocs.io, accessed on 1 March 2023), a Python library proposed to standardize the extraction and computation of biomarkers from medical images [4]. The Python version utilized in our experiments is 3.9.15. In each VOI, shape features, first-order features, second-order features, gray level co-occurrence matrix (GLCM), gray level dependence matrix (GLDM), gray level size zone matrix (GLSZM), gray level run length matrix (GLRLM), and neighboring gray-tone difference matrix (NGTDM), were extracted.

**Figure 1:**
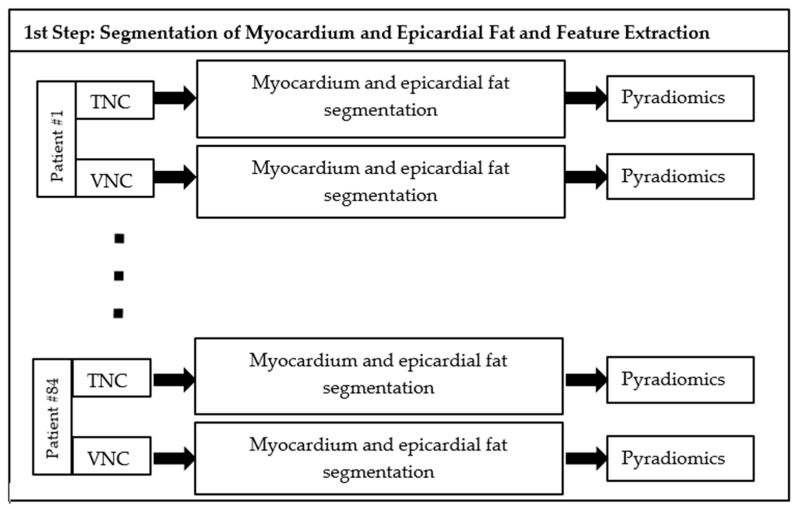
Scheme of the first step of the proposed method. For each patient (#1…#84), radiomics features are extracted in each VOI segmented in TNC and VNC reconstructions.

A summary of the proposed approach is available in Figure 1.

### 2.5. Statistical Analysis

The stability of the radiomics features between VNC and TNC was analyzed by using the intraclass correlation coefficient [21], as defined [22]. The library psych (version 2.9.9) available in R (version 4.2.2) has been used to compute the ICC coefficient. ICC3 estimate has been utilized since two fixed CT reconstructions were used to analyze the results. To compute per-feature ICC in each anatomical structure, the values of the same feature extracted in the VNC and TNC reconstructions of all patients are used as input. Thus, the number of extracted ICC values in each type of VOI equals the number of features. The features with an ICC value higher than 0.75 are considered stable (“good reliability”) [23].

**Figure 2:**
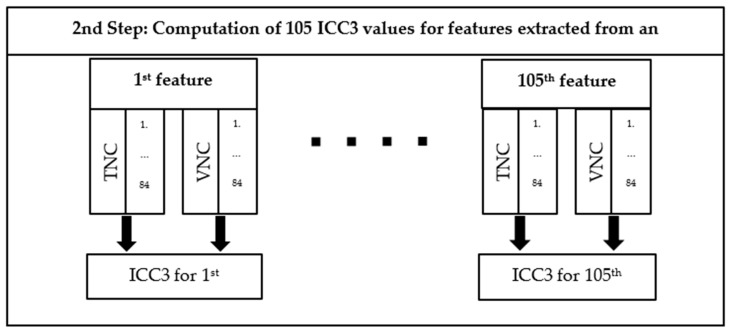
Scheme of the second step of the proposed method. For each feature, a pair of vectors is built, each of them containing 84 values of the same feature extracted respectively in each type of VOI from TNC and VNC reconstructions of all the patients. An ICC3 value is computed for each of the 105 features for each type of VOI.

A scheme of the second step of the proposed step is available in Figure 2. The computation of inter-feature correlation within each reconstruction and each anatomical structure is also conducted. The Spearman correlation of each feature against all the others is computed in R using the library stats (4.2.2). The results are provided in heat maps. The comparison of the heat maps gives another insight into the distribution of features computed in the two reconstructions for each anatomical structure.

## 3. Results

### 3.1. Patient Characteristics

The final study cohort comprises 84 patients (median age 80 years, 48 female). In the non-contrast series, the dose length product (DLP) and volumetric CT dose index (CTDIvol) were 29.4 (22.3–41.9) mGy∙cm and 1.4 (1.1–2.1) mGy, respectively. The size-specific dose estimate (SSDE) was 1.9 mGy (1.6–2.4 mGy). For contrast-enhanced acquisitions (CTA), the DLP and CTDIvol were 442.0 (329.0–583.0) mGy∙cm and 26.6 (19.9–36.7) mGy, respectively. The SSDE was 35.7 mGy (28.8–47.1 mGy). Table 1 summarizes the main characteristics of the final cohort.

### 3.2. Radiomics Feature Analysis

**Figure 3:**
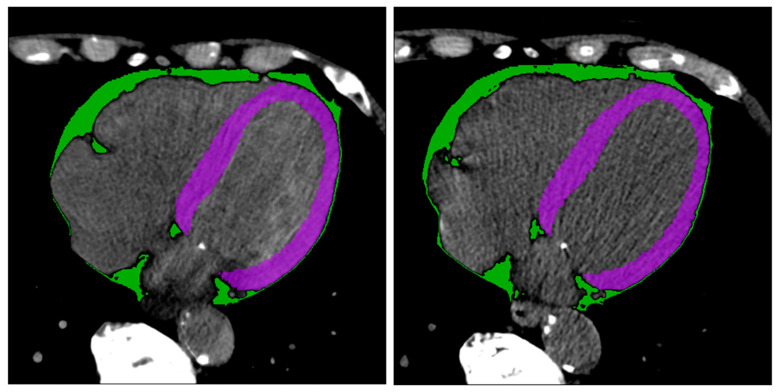
Segmentation results. Segmentation of myocardium (in green) and epicardial fat (in purple) in intra-patient TNC and VNC images (first and second subfigure, respectively). Figures are visualized with window center 97 and window width equal to 215.

**Figure 4:**
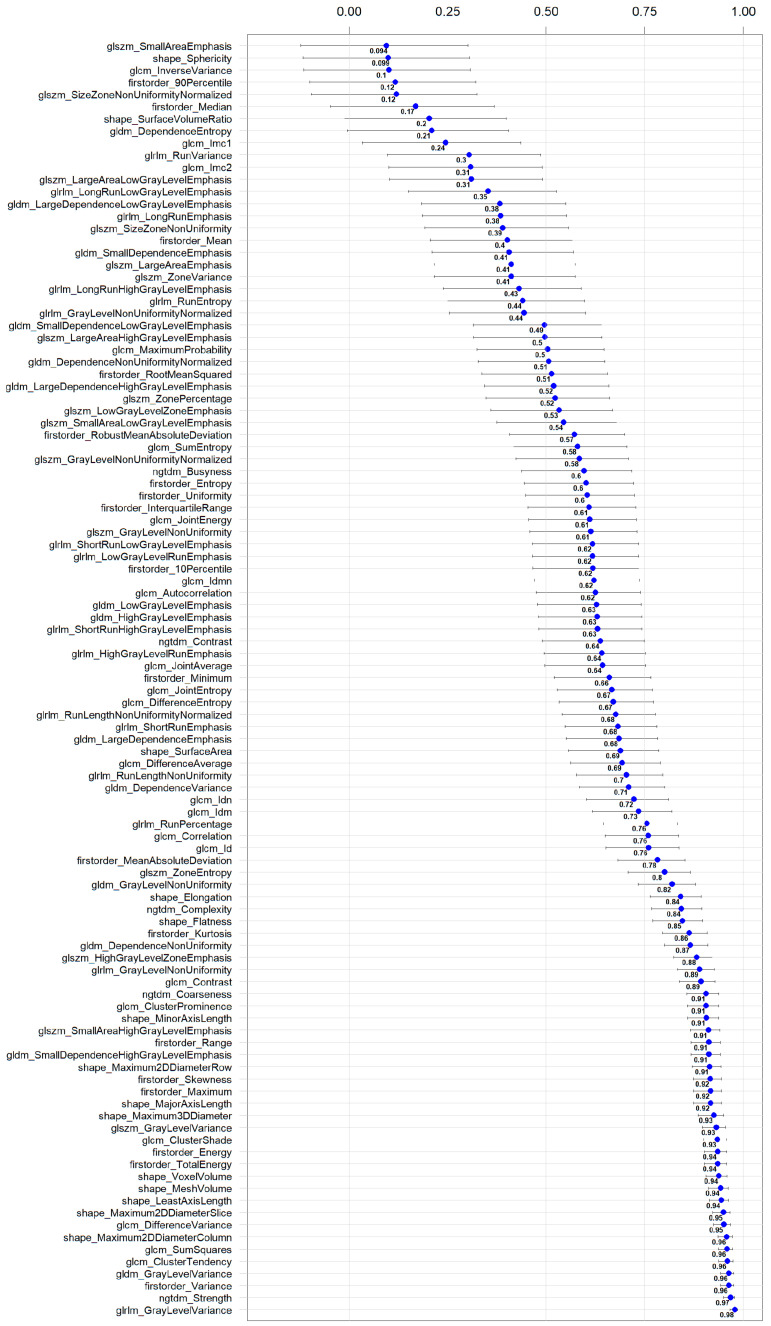
Ordered ICC values for the radiomics features extracted in the myocardium in VNC and TNC reconstructions. For each feature, the ICC coefficient is visually shown in blue, and its numerical value is reported next to it; the horizontal lines represent the 95% confidence interval.

**Figure 5:**
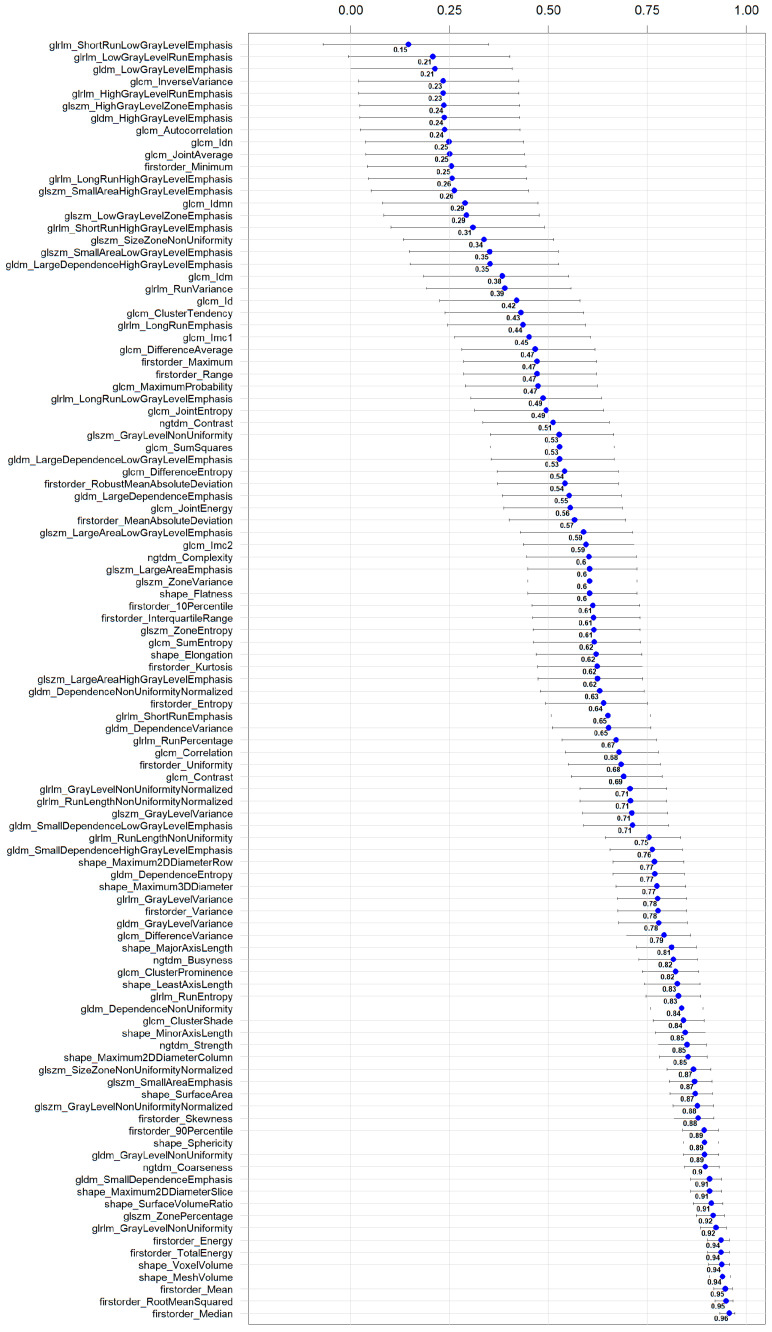
**Ordered ICC values for the radiomics features extracted in epicardial fat in VNC and TNC reconstructions.** For each feature, the ICC coefficient is visually shown in blue, and its numerical value is reported next to it; the horizontal lines represent the 95% confidence interval.

Figure 3 shows two example masks of the myocardium and epicardial fat in intra-patient TNC and VNC images. The number of radiomics features extracted in each reconstruction for every anatomical structure is 105. The complete list of extracted features is available in Figure 4 (x-axis). ICC values computed for VNC and TNC reconstructions of myocardium and epicardial fat are available in Figure 4 and Figure 5. The values of the ICC coefficients are ordered in a descending fashion to better comprehend which features are more correlated (i.e., remain more stable) between VNC and TNC acquisitions. In each Figure, the blue square identifies the related ICC value, which is also reported, and the extremities of vertical lines crossing each square represent the lower and upper bounds. Figure 4 presents the ICC values computed for the features extracted from the myocardium. Similarly, Figure 5 displays the ordered ICC values computed for the features extracted for the epicardial fat.

A total of 24 features remained stable across both anatomical structures, the myocardium and epicardial fat, after analysis. The names of these stable features are listed in Table 2.

Stable features in the myocardium are 41, and the three with the highest ICC are glrlm_GrayLevelVariance 0.98 [0.97, 0.99], ngtdm_Strength 0.97 [0.95, 0.98], firstorder_Variance 0.96 [0.94, 0.98]. For the epicardial fat, stable features are 40, and the three highest ranked are firstorder_Median 0.96 [0.93, 0.97], firstorder_RootMeanSquared 0.95 [0.92, 0.97], firstorder_Mean 0.95 [0.92, 0.97]. Additional detailed information for 105 features, categorized by anatomical structure (epicardial fat and myocardium), is provided in separate tables within the Supplementary Document as Supplemental Tables S1 and S2.

Supplemental Figures S1 and S2 present each of the two heat maps showing the inter-feature correlation for myocardium and epicardial fat for the features extracted for VNC and TNC reconstructions, respectively. The detailed visual representation of the data is provided as extra information in another document. All heatmaps are presented in the “Supplementary Document”. This approach was employed to make the research easier to read by displaying the complex visual data in a form that is clear and understandable.

## 4. Discussion

This study investigated the interchangeability of the TNC and VNC series by correlating Pyradiomics features in EAT and myocardium. We found that there is a high variance between TNC and VNC radiomics, which could affect the effectiveness of image-based methods. In both TNC and VNC, about 40% of features showed good reliability, while only about 23% of features (24/105) achieved this level of correlation in VNC and TNC for both EAT and the myocardium.

Texture analysis based on radiomics features is a promising approach for improving how we identify and understand medical images, with the goal of creating valuable predictive or prognostic biomarkers. However, the variability that comes with different image acquisition techniques can affect the consistency of pixel or voxel values, which might compromise the reliability of texture analysis [24]. Thus, it is important to quantitatively evaluate how stable or repeatable these texture features are, especially when comparing VNC and TNC imaging [25]. In this regard, there is still a critical gap in understanding how these two reconstructions are interchangeable, underscoring the need for more research to ensure that texture analysis can be both dependable and consistent across various imaging techniques.

Recent research has highlighted the clinical value of using VNC cardiac imaging as an alternative to TNC imaging. This is particularly true for evaluating EAT. A 2022 study found that VNC reconstructions from photon-counting computed tomography angiography (PCD-CCTA) datasets provide accurate measurements of EAT volume, with results closely aligned to those obtained from TNC images. This approach can significantly lower patient radiation exposure while maintaining high accuracy in CT value assessments [26]. Another research from our clinic involved 42 patients and demonstrated that VNCPC reconstructions of PCD-CCTA datasets can accurately measure EAT volume, with only slight differences in CT values compared to TNC. Using VNCPC instead of TNC could significantly reduce the radiation exposure of patients. Based on these results, our analysis also takes into consideration VNC volumes reconstructed by using the Pure Calcium algorithm since it obtains better performances than other algorithms [12].

CT-based extracellular volume (ECV) measurement has proven effective in assessing myocardial health, especially for conditions like cardiac amyloidosis and fibrosis following a myocardial infarction [27]. VNC series of the heart could potentially replace the TNC series for assessing ECV in the myocardium by preserving diagnostic precision and significantly decreasing patient radiation exposure. In a recent study, the authors utilized radiomics features to compare different reconstructions obtained by spectral imaging dataset obtained for an organic phantom and a cohort of 23 patients with PCD-CT scanners [2]. Furthermore, a different study analyzed whether myocardial texture changes can be identified by texture analysis depending on the severity of coronary artery calcification in photon counting reconstructions [5]. Another report highlighted that the authors compared features of interpatient acquisitions obtained with energy-integrating and photon-counting scanners [6]. In another study published in 2024, EAT radiomics features of 52 patients undergoing PCCT were quantified using images reconstructed with VNCPC, series generated with another conventional reconstruction algorithm (VNCConv), and TNC data. This study showed that VNCPC and VNCConv tend to underestimate EATVs and overestimate EATDs [28]. The main difference between our study and previous research from 2024 is that we examine both EAT and the myocardium. Furthermore, our findings are based on approximately 60% more patients [28], enhancing the validity and applicability of the results [29].

Despite this, the current study has some limitations. First, this is a single-centric retrospectively collected cohort of 84 patients, with 105 features evaluated, of which 24 exhibited stability (ICC > 0.75) in both volumes. In comparison to prior human radiomic studies, our sample size may be smaller, and the number of evaluated features is relatively high, which could influence the power and generalizability of our findings. Larger cohorts may yield more robust insights into feature stability. Second, only VNC pure calcium (VNCPC) reconstructions were used, and no conventional VNC algorithms were used. However, multiple studies showed higher suitability for this novel VNC algorithm to replace TNC [30,31,32], which is why we chose it for this comparison. Third, there are significant differences in the acquisition parameters, particularly the considerable discrepancy in radiation dose between the TNC and CTA protocols from which the VNC was generated, which likely contributes to the observed differences in radiomics features. This is because TNC is a low-dose, high-pitch scan designed for coronary calcium scoring. However, our results show that TNC and VNC may differ more than can be observed at first glance or by the human eye. This raises the critical point that a simple substitution of TNC with VNC may be not only challenging for radiomics studies but also for clinical routine. Further studies are necessary to investigate this, emphasizing the clinical relevance of our findings, as the observed differences are significant. In clinical routine and VNC research, the TNC series should be replaced by the VNC series generated from CTA acquisitions. Therefore, from a clinical point of view, we chose this series to stay as close as possible to clinically present scenarios. Finally, no phantom imaging studies were included to validate the system’s calibration or ensure that the radiomics features are comparable across different acquisition protocols. Future studies should incorporate phantom imaging to assess the stability of radiomics features under varying conditions and improve the robustness and applicability of the findings.

## 5. Conclusions

In conclusion, while VNC reconstructions derived from cardiac PCD-CT datasets show promise for reducing patient radiation exposure and maintaining diagnostic accuracy, the variability in radiomics feature stability between TNC and VNC series in the myocardium and the epicardial adipose tissue series indicates that further research is needed to optimize these techniques for texture assessment to ensure the reliability of image-based analyses.

## Figures and Tables

**Table 1:** Baseline study characteristics.

Final Cohort = 84 Patients		
Age, years	80 (75–84)	
Sex, female	48/84 (57.1%)	
CT acquisitions	Non-contrast (TNC)	CTA
CTDIvol (mGy)	1.4 (1.1–2.1)	26.6 (19.9–36.7)
DLP (mGy∙cm)	29.4 (22.3–41.9)	442.0 (329.0–583.0)
SSDE (mGy)	1.9 (1.6–2.4)	35.7 (28.8–47.1)

Data are presented as median value (with interquartile range) or percentage. CT computed tomography, SSDE size-specific dose estimate, DLP dose length product, CTDIvol volumetric CT dose index.

**Table 2:** List of Stable Features between epicardial adipose tissue and the myocardium.

Stable Features Between Two Structures	Epicardial Adipose Tissue	Myocardium
shape_MeshVolume	0.94 (0.91, 0.96)	0.94 (0.91, 0.96)
shape_VoxelVolume	0.94 (0.91, 0.96)	0.94 (0.91, 0.96)
firstorder_Energy	0.94 (0.9, 0.96)	0.94 (0.9, 0.96)
firstorder_TotalEnergy	0.94 (0.9, 0.96)	0.94 (0.9, 0.96)
glrlm_GrayLevelNonUniformity	0.92 (0.88, 0.95)	0.89 (0.83, 0.93)
shape_Maximum2DDiameterSlice	0.91 (0.86, 0.94)	0.95 (0.92, 0.97)
ngtdm_Coarseness	0.9 (0.84, 0.93)	0.91 (0.86, 0.94)
gldm_GrayLevelNonUniformity	0.89 (0.84, 0.93)	0.82 (0.73, 0.88)
firstorder_Skewness	0.88 (0.82, 0.92)	0.92 (0.87, 0.94)
ngtdm_Strength	0.85 (0.78, 0.9)	0.97 (0.95, 0.98)
shape_Maximum2DDiameterColumn	0.85 (0.78, 0.9)	0.96 (0.94, 0.97)
shape_MinorAxisLength	0.85 (0.77, 0.9)	0.91 (0.86, 0.94)
glcm_ClusterShade	0.84 (0.76, 0.89)	0.93 (0.9, 0.96)
gldm_DependenceNonUniformity	0.84 (0.76, 0.89)	0.87 (0.8, 0.91)
shape_LeastAxisLength	0.83 (0.74, 0.88)	0.94 (0.91, 0.96)
glcm_ClusterProminence	0.82 (0.74, 0.88)	0.91 (0.86, 0.94)
shape_MajorAxisLength	0.81 (0.72, 0.87)	0.92 (0.87, 0.95)
glcm_DifferenceVariance	0.79 (0.7, 0.86)	0.95 (0.93, 0.97)
glrlm_GrayLevelVariance	0.78 (0.67, 0.85)	0.98 (0.97, 0.99)
gldm_GrayLevelVariance	0.78 (0.68, 0.85)	0.96 (0.94, 0.98)
firstorder_Variance	0.78 (0.68, 0.85)	0.96 (0.94, 0.98)
shape_Maximum3DDiameter	0.77 (0.67, 0.85)	0.93 (0.89, 0.95)
shape_Maximum2DDiameterRow	0.77 (0.66, 0.84)	0.91 (0.87, 0.94)
gldm_SmallDependenceHighGrayLevelEmphasis	0.76 (0.66, 0.84)	0.91 (0.87, 0.94)

Table sorted in descending ordered by epicardial adipose tissue. Data are presented as the calculated ICC value with the corresponding 95% confidence interval.

## Data Availability

The data presented in this study are available on request from the corresponding author.

## Supplementary Materials

The following supporting information can be downloaded at https://www.mdpi.com/article/10.3390/diagnostics14222483/s1, Figure S1: Heatmaps showing the Spearman correlation values computed between each feature extracted in epicardial fat. The first Figure is about the radiomics features extracted in VNC reconstructions. Figure S2: Heatmaps showing the Spearman correlation values computed between each feature extracted in epicardial fat. The first Figure is about the radiomics features extracted in TNC reconstructions. Table S1: Myocardium-ICC Sorted Data. Table S2: Epicardial Fat-ICC Sorted Data.
