# Supplementary material for: Radiomics Feature Stability in True and Virtual Non-Contrast Reconstructions from Cardiac Photon-Counting Detector CT Datasets
Source: Diagnostics (Basel). 2024 Nov 7;14(22):2483. doi: 10.3390/diagnostics14222483 (PMC11592515; doi:10.3390/diagnostics14222483)
Supplement: Supplementary file 1 [file diagnostics-14-02483-s001.zip › diagnostics-3254004-supplementary.pdf]

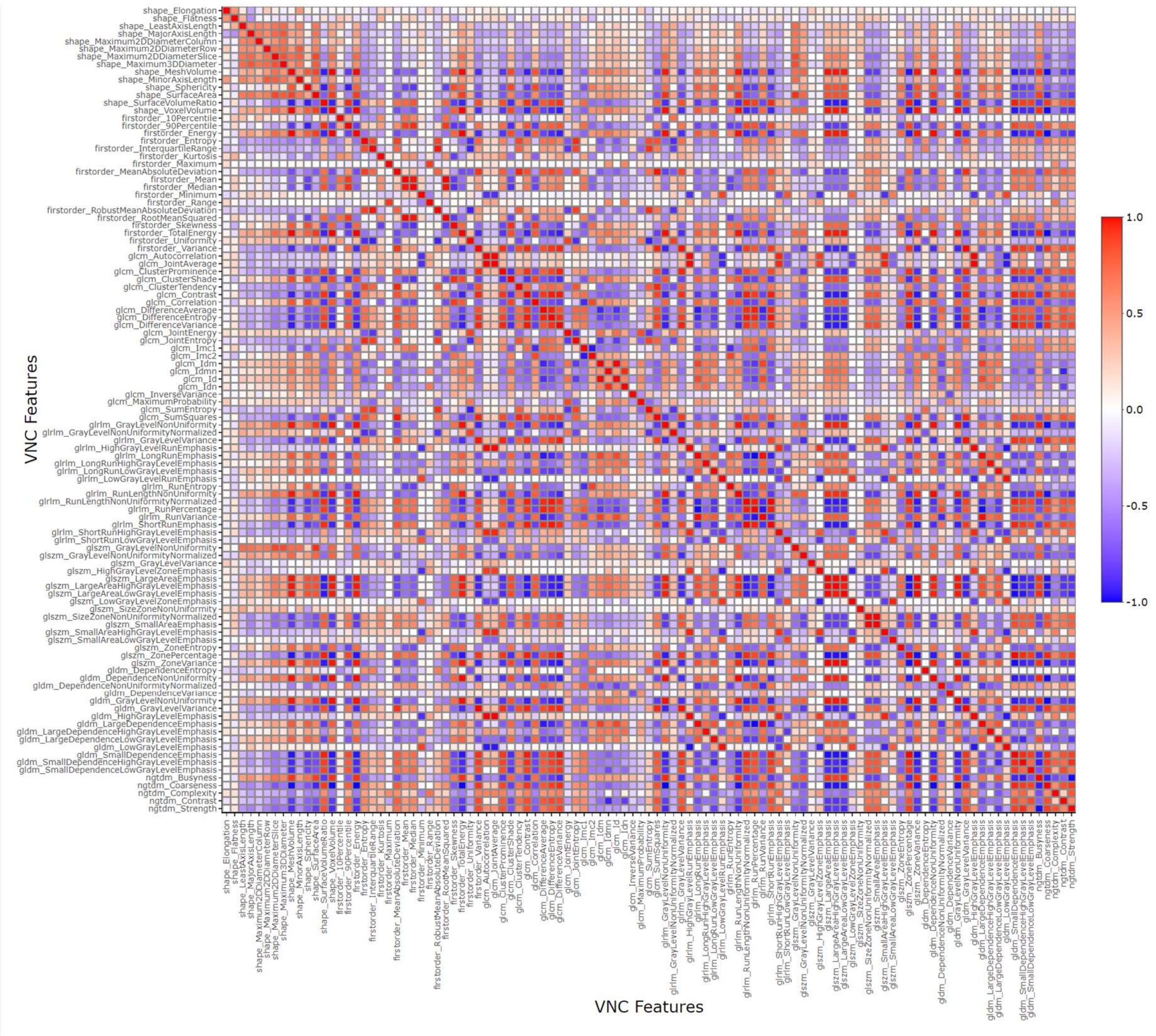

Supplemental Figure S1 Heatmaps showing the Spearman correlation values computed between each feature extracted in epicardial fat. The first figure is about the radiomics features extracted in VNC reconstructions.

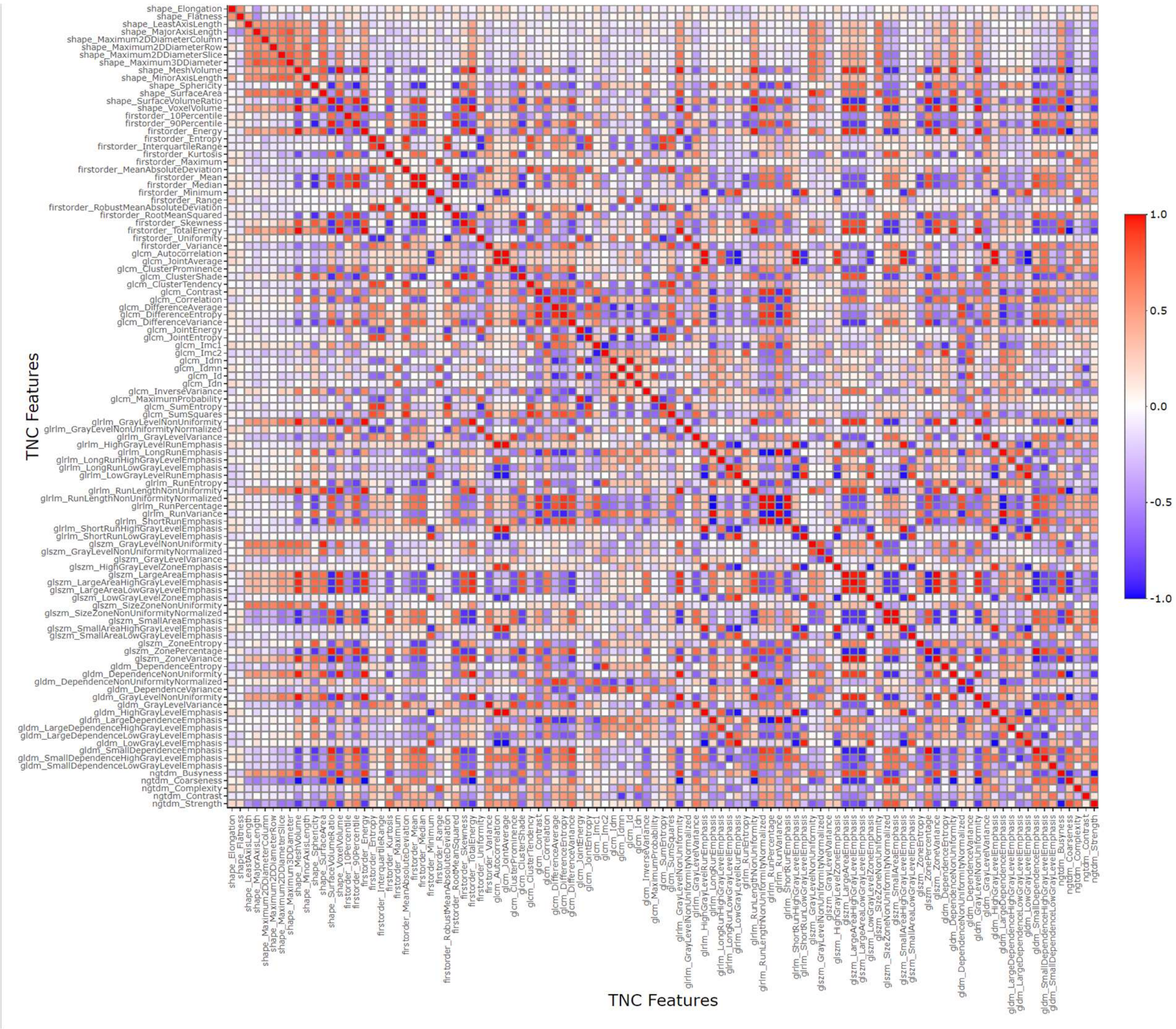

Supplemental Figure S2 Heatmaps showing the Spearman correlation values computed between each feature extracted in epicardial fat. The first figure is about the radiomics features extracted in TNC reconstructions.

Supplemental Table S1: Myocardium-ICC Sorted Data

Feature	ICC3 95% CI
glrlm_GrayLevelVariance	0.98 (0.97, 0.99)
ngtdm_Strength	0.97 (0.95, 0.98)
firstorder_Variance	0.96 (0.94, 0.98)
gldm_GrayLevelVariance	0.96 (0.94, 0.98)
glcm_ClusterTendency	0.96 (0.94, 0.97)
glcm_SumSquares	0.96 (0.94, 0.97)
shape_Maximum2DDiameterColumn	0.96 (0.94, 0.97)
glcm_DifferenceVariance	0.95 (0.93, 0.97)
shape_Maximum2DDiameterSlice	0.95 (0.92, 0.97)
shape_LeastAxisLength	0.94 (0.91, 0.96)
shape_MeshVolume	0.94 (0.91, 0.96)
shape_VoxelVolume	0.94 (0.91, 0.96)
firstorder_TotalEnergy	0.94 (0.9, 0.96)
firstorder_Energy	0.94 (0.9, 0.96)
glcm_ClusterShade	0.93 (0.9, 0.96)
glszm_GrayLevelVariance	0.93 (0.9, 0.95)
shape_Maximum3DDiameter	0.93 (0.89, 0.95)
shape_MajorAxisLength	0.92 (0.87, 0.95)
firstorder_Maximum	0.92 (0.87, 0.95)
firstorder_Skewness	0.92 (0.87, 0.94)
shape_Maximum2DDiameterRow	0.91 (0.87, 0.94)
gldm_SmallDependenceHighGrayLevelEmphasis	0.91 (0.87, 0.94)
firstorder_Range	0.91 (0.87, 0.94)
glszm_SmallAreaHighGrayLevelEmphasis	0.91 (0.87, 0.94)
shape_MinorAxisLength	0.91 (0.86, 0.94)
glcm_ClusterProminence	0.91 (0.86, 0.94)
ngtdm_Coarseness	0.91 (0.86, 0.94)
glcm_Contrast	0.89 (0.84, 0.93)
glrlm_GrayLevelNonUniformity	0.89 (0.83, 0.93)
glszm_HighGrayLevelZoneEmphasis	0.88 (0.82, 0.92)
gldm_DependenceNonUniformity	0.87 (0.8, 0.91)
firstorder_Kurtosis	0.86 (0.8, 0.91)
shape_Flatness	0.85 (0.77, 0.9)
ngtdm_Complexity	0.84 (0.77, 0.9)
shape_Elongation	0.84 (0.76, 0.89)
gldm_GrayLevelNonUniformity	0.82 (0.73, 0.88)
glszm_ZoneEntropy	0.8 (0.71, 0.87)
firstorder_MeanAbsoluteDeviation	0.78 (0.68, 0.85)
glcm_Id	0.76 (0.65, 0.84)
glcm_Correlation	0.76 (0.65, 0.84)
glrlm_RunPercentage	0.76 (0.65, 0.83)
glcm_Idm	0.73 (0.62, 0.82)
glcm_Idn	0.72 (0.6, 0.81)

gldm_DependenceVariance	0.71 (0.58, 0.8)
glrlm_RunLengthNonUniformity	0.7 (0.58, 0.8)
glcm_DifferenceAverage	0.69 (0.56, 0.79)
shape_SurfaceArea	0.69 (0.56, 0.79)
gldm_LargeDependenceEmphasis	0.68 (0.55, 0.78)
glrlm_ShortRunEmphasis	0.68 (0.55, 0.78)
glrlm_RunLengthNonUniformityNormalized	0.68 (0.54, 0.78)
glcm_DifferenceEntropy	0.67 (0.53, 0.77)
glcm_JointEntropy	0.67 (0.53, 0.77)
firstorder_Minimum	0.66 (0.52, 0.77)
glcm_JointAverage	0.64 (0.5, 0.75)
glrlm_HighGrayLevelRunEmphasis	0.64 (0.5, 0.75)
ngtdm_Contrast	0.64 (0.49, 0.75)
glrlm_ShortRunHighGrayLevelEmphasis	0.63 (0.48, 0.74)
gldm_HighGrayLevelEmphasis	0.63 (0.48, 0.74)
gldm_LowGrayLevelEmphasis	0.63 (0.48, 0.74)
glcm_Autocorrelation	0.62 (0.47, 0.74)
glcm_Idmn	0.62 (0.47, 0.74)
firstorder_10Percentile	0.62 (0.47, 0.73)
glrlm_LowGrayLevelRunEmphasis	0.62 (0.47, 0.73)
glrlm_ShortRunLowGrayLevelEmphasis	0.62 (0.47, 0.73)
glszm_GrayLevelNonUniformity	0.61 (0.46, 0.73)
glcm_JointEnergy	0.61 (0.46, 0.73)
firstorder_InterquartileRange	0.61 (0.45, 0.73)
firstorder_Uniformity	0.6 (0.45, 0.72)
firstorder_Entropy	0.6 (0.44, 0.72)
ngtdm_Busyness	0.6 (0.44, 0.72)
glszm_GrayLevelNonUniformityNormalized	0.58 (0.42, 0.71)
glcm_SumEntropy	0.58 (0.42, 0.71)
firstorder_RobustMeanAbsoluteDeviation	0.57 (0.41, 0.7)
glszm_SmallAreaLowGrayLevelEmphasis	0.54 (0.37, 0.68)
glszm_LowGrayLevelZoneEmphasis	0.53 (0.36, 0.67)
glszm_ZonePercentage	0.52 (0.35, 0.66)
gldm_LargeDependenceHighGrayLevelEmphasis	0.52 (0.34, 0.66)
firstorder_RootMeanSquared	0.51 (0.34, 0.65)
gldm_DependenceNonUniformityNormalized	0.51 (0.33, 0.65)
glcm_MaximumProbability	0.5 (0.33, 0.65)
glszm_LargeAreaHighGrayLevelEmphasis	0.5 (0.32, 0.64)
gldm_SmallDependenceLowGrayLevelEmphasis	0.49 (0.31, 0.64)
glrlm_GrayLevelNonUniformityNormalized	0.44 (0.25, 0.6)
glrlm_RunEntropy	0.44 (0.25, 0.6)
glrlm_LongRunHighGrayLevelEmphasis	0.43 (0.24, 0.59)
glszm_ZoneVariance	0.41 (0.22, 0.57)
glszm_LargeAreaEmphasis	0.41 (0.22, 0.57)
gldm_SmallDependenceEmphasis	0.41 (0.21, 0.57)
firstorder_Mean	0.4 (0.21, 0.57)

glszm_SizeZoneNonUniformity	0.39 (0.19, 0.56)
glrlm_LongRunEmphasis	0.38 (0.19, 0.55)
gldm_LargeDependenceLowGrayLevelEmphasis	0.38 (0.18, 0.55)
glrlm_LongRunLowGrayLevelEmphasis	0.35 (0.15, 0.53)
glszm_LargeAreaLowGrayLevelEmphasis	0.31 (0.1, 0.49)
glcm_Imc2	0.31 (0.1, 0.49)
glrlm_RunVariance	0.3 (0.1, 0.49)
glcm_Imc1	0.24 (0.03, 0.44)
gldm_DependenceEntropy	0.21 (-0.0, 0.4)
shape_SurfaceVolumeRatio	0.2 (-0.01, 0.4)
firstorder_Median	0.17 (-0.05, 0.37)
glszm_SizeZoneNonUniformityNormalized	0.12 (-0.1, 0.32)
firstorder_90Percentile	0.12 (-0.1, 0.32)
glcm_InverseVariance	0.1 (-0.11, 0.31)
shape_Sphericity	0.1 (-0.12, 0.31)
glszm_SmallAreaEmphasis	0.09 (-0.12, 0.3)

Supplemental Table S2: Epicardial Fat-ICC Sorted Data

Feature	ICC3 95% CI
firstorder_Median	0.96 (0.93, 0.97)
firstorder_RootMeanSquared	0.95 (0.92, 0.97)
firstorder_Mean	0.95 (0.92, 0.97)
shape_MeshVolume	0.94 (0.91, 0.96)
shape_VoxelVolume	0.94 (0.91, 0.96)
firstorder_TotalEnergy	0.94 (0.9, 0.96)
firstorder_Energy	0.94 (0.9, 0.96)
glrlm_GrayLevelNonUniformity	0.92 (0.88, 0.95)
glszm_ZonePercentage	0.92 (0.87, 0.94)
shape_SurfaceVolumeRatio	0.91 (0.87, 0.94)
shape_Maximum2DDiameterSlice	0.91 (0.86, 0.94)
gldm_SmallDependenceEmphasis	0.91 (0.86, 0.94)
ngtdm_Coarseness	0.9 (0.84, 0.93)
gldm_GrayLevelNonUniformity	0.89 (0.84, 0.93)
shape_Sphericity	0.89 (0.84, 0.93)
firstorder_90Percentile	0.89 (0.84, 0.93)
firstorder_Skewness	0.88 (0.82, 0.92)
glszm_GrayLevelNonUniformityNormalized	0.88 (0.81, 0.92)
shape_SurfaceArea	0.87 (0.81, 0.91)
glszm_SmallAreaEmphasis	0.87 (0.81, 0.91)
glszm_SizeZoneNonUniformityNormalized	0.87 (0.8, 0.91)
shape_Maximum2DDiameterColumn	0.85 (0.78, 0.9)
ngtdm_Strength	0.85 (0.78, 0.9)
shape_MinorAxisLength	0.85 (0.77, 0.9)
glcm_ClusterShade	0.84 (0.76, 0.89)
gldm_DependenceNonUniformity	0.84 (0.76, 0.89)
girlm_RunEntropy	0.83 (0.75, 0.89)
shape_LeastAxisLength	0.83 (0.74, 0.88)
glcm_ClusterProminence	0.82 (0.74, 0.88)
ngtdm_Busyness	0.82 (0.73, 0.88)
shape_MajorAxisLength	0.81 (0.72, 0.87)
glcm_DifferenceVariance	0.79 (0.7, 0.86)
gldm_GrayLevelVariance	0.78 (0.68, 0.85)
firstorder_Variance	0.78 (0.68, 0.85)
glrlm_GrayLevelVariance	0.78 (0.67, 0.85)
shape_Maximum3DDiameter	0.77 (0.67, 0.85)
gldm_DependenceEntropy	0.77 (0.66, 0.84)
shape_Maximum2DDiameterRow	0.77 (0.66, 0.84)
gldm_SmallDependenceHighGrayLevelEmphasis	0.76 (0.66, 0.84)
glrim_RunLengthNonUniformity	0.75 (0.64, 0.83)
gldm_SmallDependenceLowGrayLevel&mphasis	0.71 (0.59, 0.8)
glszm_GrayLevelVariance	0.71 (0.59, 0.8)
glrlm_RunLengthNonUniformityNormalized	0.71 (0.58, 0.8)

glrlm_GrayLevelNonUniformityNormalized	0.71 (0.58, 0.8)
glcm_Contrast	0.69 (0.56, 0.79)
firstorder_Uniformity	0.68 (0.55, 0.78)
glcm_Correlation	0.68 (0.54, 0.78)
glrlm_RunPercentage	0.67 (0.53, 0.77)
gldm_DependenceVariance	0.65 (0.51, 0.76)
glrlm_ShortRunEmphasis	0.65 (0.51, 0.76)
firstorder_Entropy	0.64 (0.49, 0.75)
gldm_DependenceNonUniformityNormalized	0.63 (0.48, 0.74)
glszm_LargeAreaHighGrayLevelEmphasis	0.62 (0.47, 0.74)
firstorder_Kurtosis	0.62 (0.47, 0.74)
shape_Elongation	0.62 (0.47, 0.74)
glcm_SumEntropy	0.62 (0.46, 0.73)
glszm_ZoneEntropy	0.61 (0.46, 0.73)
firstorder_InterquartileRange	0.61 (0.46, 0.73)
firstorder_10Percentile	0.61 (0.46, 0.73)
shape_Flatness	0.6 (0.45, 0.72)
glszm_ZoneVariance	0.6 (0.45, 0.72)
glszm_LargeAreaEmphasis	0.6 (0.45, 0.72)
ngtdm_Complexity	0.6 (0.45, 0.72)
glcm_Imc2	0.59 (0.44, 0.72)
glszm_LargeAreaLowGrayLevelEmphasis	0.59 (0.43, 0.71)
firstorder_MeanAbsoluteDeviation	0.57 (0.4, 0.7)
glcm_JointEnergy	0.56 (0.39, 0.69)
gldm_LargeDependenceEmphasis	0.55 (0.38, 0.68)
firstorder_RobustMeanAbsoluteDeviation	0.54 (0.37, 0.68)
glcm_DifferenceEntropy	0.54 (0.37, 0.68)
gldm_LargeDependenceLowGrayLevelEmphasis	0.53 (0.35, 0.67)
glcm_SumSquares	0.53 (0.35, 0.67)
glszm_GrayLevelNonUniformity	0.53 (0.35, 0.67)
ngtdm_Contrast	0.51 (0.33, 0.65)
glcm_JointEntropy	0.49 (0.31, 0.64)
glrlm_LongRunLowGrayLevelEmphasis	0.49 (0.3, 0.63)
glcm_MaximumProbability	0.47 (0.29, 0.62)
firstorder_Range	0.47 (0.29, 0.62)
firstorder_Maximum	0.47 (0.29, 0.62)
glcm_DifferenceAverage	0.47 (0.28, 0.62)
glcm_Imc1	0.45 (0.26, 0.61)
glrlm_LongRunEmphasis	0.44 (0.25, 0.59)
glcm_ClusterTendency	0.43 (0.24, 0.59)
glcm_Id	0.42 (0.23, 0.58)
glrlm_RunVariance	0.39 (0.19, 0.56)
glcm_Idm	0.38 (0.19, 0.55)
gldm_LargeDependenceHighGrayLevelEmphasis	0.35 (0.15, 0.53)
glszm_SmallAreaLowGrayLevelEmphasis,	0.35 (0.15, 0.52)
glszm_SizeZoneNonUniformity	0.34 (0.13, 0.51)

glrlm_ShortRunHighGrayLevelEmphasis	0.31 (0.1, 0.49)
glszm_LowGrayLevelZoneEmphasis	0.29 (0.08, 0.48)
glcm_Idmn	0.29 (0.08, 0.47)
glszm_SmallAreaHighGrayLevelEmphasis	0.26 (0.05, 0.45)
glrlm_LongRunHighGrayLevelEmphasis	0.26 (0.05, 0.45)
firstorder_Minimum	0.25 (0.04, 0.44)
glcm_JointAverage	0.25 (0.04, 0.44)
glcm_Idn	0.25 (0.04, 0.44)
glcm_Autocorrelation	0.24 (0.02, 0.43)
gldm_HighGrayLevelEmphasis	0.24 (0.02, 0.43)
glszm_HighGrayLevelZoneEmphasis	0.24 (0.02, 0.43)
glrlm_HighGrayLevelRunEmphasis	0.23 (0.02, 0.43)
glcm_InverseVariance	0.23 (0.02, 0.43)
gldm_LowGrayLevelEmphasis	0.21 (-0.0, 0.41)
glrlm_LowGrayLevelRunEmphasis	0.21 (-0.01, 0.4)
glrlm_ShortRunLowGrayLevelEmphasis	0.15 (-0.07, 0.35)
